# MINOCA y Ectasia Coronaria : una combinación inusual de causas de infarto

**DOI:** 10.31053/1853.0605.v81.n1.43231

**Published:** 2024-03-27

**Authors:** Juan Pablo Ricarte-Bratti, Julio Oscar Emilio Bono, Matias Morsone, Hugo jose Londero

**Affiliations:** 1 Sanatorio Allende, Unidad Cardiovascular Córdoba Argentina; 2 Universidad Nacional de Córdoba. Facultad de Ciencias Médicas. Cátedra de Farmacología Córdoba Argentina; 3 Sanatorio Allende. Servicio de Hemodinamia, angiografía y cardiología intervencionista Córdoba Argentina

**Keywords:** infarto de miocardio con arterias coronarias no obstructivas (MINOCA), isquemia miocárdica, infarto de miocardio, myocardial infarction with non-obstructive coronary arteries (MINOCA), myocardial ischemia, myocardial infarction, infarto do miocárdio com artérias coronárias não obstrutivas (MINOCA), isquemia miocárdica, infarto do miocárdio

## Abstract

**Objetivo:**

describir un paciente con isquemia miocárdica con múltiples causas.

**Caso Clínico:**

En este caso clínico se describe a un hombre de 58 años con antecedentes de hipertensión, dislipidemia, EPOC e infarto de miocardio (IAM) previo. Acudió a urgencias con dolor torácico y disnea. Los hallazgos incluyeron crepitantes bibasales, electrocardiograma con fibrosis anterior antigua, NT-ProBNP elevado y ecocardiograma con acinesia septoapical. Durante la hospitalización, experimentó taquiarritmia y deterioro hemodinámico, siendo sometido a cardioversión eléctrica (CVE). Se diagnosticó síndrome coronario agudo sin elevación del segmento ST (SCASEST) complicado con arritmia ventricular y edema pulmonar agudo. La angiografía coronaria reveló ectasias coronarias sin lesiones obstructivas, pero con estenosis leve en tres vasos. El paciente fue tratado con éxito mediante ventilación no invasiva, diuréticos, vasodilatadores y anticoagulación. Se otorgó el alta con el plan de profundizar estudios para optimizar y guiar tratamiento y finalmente se abordó al diagnóstico de Infarto de Miocardio con Arterias No Obstructivas (MINOCA) y la presencia de ectasias coronarias.

**Conclusión:**

es importante destacar las causas no isquémicas en MINOCA y la asociación entre ectasia coronaria y eventos cardiovasculares, por lo que subrayamos la necesidad de más estudios para comprender mejor la relación entre estos fenómenos.

## Abstract (en)

**Objective:**

to describe a patient with myocardial ischemia with multiple causes.

**Clinical Case:**

This clinical case describes a 58-year-old man with a history of hypertension, dyslipidemia, COPD and previous myocardial infarction (AMI). He went to the emergency room with chest pain and dyspnea. Findings included bibasal crackles, electrocardiogram with old anterior fibrosis, elevated NT-ProBNP, and echocardiogram with septoapical akinesia. During hospitalization, he experienced tachyarrhythmia and hemodynamic deterioration, undergoing electrical cardioversion (CVE). Non-ST segment elevation acute coronary syndrome (NSTEACS) complicated with ventricular arrhythmia and acute pulmonary edema was diagnosed. Coronary angiography revealed coronary ectasias without obstructive lesions, but with mild stenosis in three vessels. The patient was successfully treated with non-invasive ventilation, diuretics, vasodilators and anticoagulation. The discharge was granted with the plan to further studies to optimize and guide treatment and finally the diagnosis of Myocardial Infarction with Non-Obstructive Arteries (MINOCA) and the presence of coronary ectasias was addressed.

**Conclusion:**

it is important to highlight the non-ischemic causes in MINOCA and the association between coronary ectasia and cardiovascular events, which is why we emphasize the need for more studies to better understand the relationship between these phenomena.

## Resumo

**Objetivo:**

descrever um paciente com isquemia miocárdica de múltiplas causas.

**Caso Clínico:**

No presente caso clínico, descreve-se um homem de 58 anos com histórico de hipertensão, dislipidemia, DPOC e infarto do miocárdio (IAM) prévio. Ele procurou atendimento de emergência com dor torácica e dispneia. Os achados incluíram crepitações bibasais, eletrocardiograma com fibrose anterior antiga, NT-ProBNP elevado e ecocardiograma com acinesia septoapical. Durante a hospitalização, ele apresentou taquiarritmia e deterioração hemodinâmica, sendo submetido a cardioversão elétrica (CVE). O diagnóstico foi síndrome coronariana aguda sem elevação do segmento ST (SCASEST) complicada por arritmia ventricular e edema pulmonar agudo.A angiografia coronariana revelou ectasias coronarianas sem lesões obstrutivas, mas com estenose leve em três vasos. O paciente foi tratado com sucesso com ventilação não invasiva, diuréticos, vasodilatadores e anticoagulação. A alta foi concedida com previsão de novos estudos para otimizar e orientar o tratamento e por fim foi abordado o diagnóstico de Infarto do Miocárdio com Artérias Não Obstrutivas (MINOCA) e presença de ectasias coronarianas.

**Conclusão:**

é importante destacar as causas não isquêmicas no MINOCA e a associação entre ectasia coronariana e eventos cardiovasculares, por isso enfatizamos a necessidade de mais estudos para melhor compreender a relação entre esses fenômenos.

**CONCEPTOS CLAVE**

**Qué se sabe sobre el tema.**

El MINOCA (Infarto de Miocardio con Arterias Coronarias No Obstructivas) es un tipo de infarto de miocardio causado por un mecanismo distinto al infarto convencional, demandando así un enfoque particular para su tratamiento.

**Qué aporta este trabajo.**

Nuestro caso describe cómo una infrecuente entidad, es producida en este caso por distintos mecanismos que se solapan con la consecuente disminución del aporte de oxígeno al miocardio.

**Divulgación**

El MINOCA (Infarto de Miocardio con Arterias Coronarias No Obstructivas) es un tipo de infarto que a diferencia del clásico, no se observa obstrucción significativa en las arterias coronarias durante los estudios angiográficos. Aunque el término se refiere a una condición específica, es importante destacar que MINOCA no representa una entidad homogénea, sino más bien un grupo de síndromes que comparten la característica común de la ausencia de obstrucción evidente en las arterias coronarias. En algunos casos, la etiología puede ser multifactorial por lo que su abordaje requiere una evaluación exhaustiva y personalizada para determinar la estrategia terapéutica más apropiada.

## Introducción

Este caso destaca las múltiples causas de infarto de miocardio (IM) en un paciente con IM con Arterias Coronarias No Obstructivas (MINOCA) y la vinculación con la Ectasia de arterias Coronarias (EAC), las implicaciones diagnósticas, pronosticas y terapéuticas.

## Caso Clínico

Un hombre de 58 años con antecedentes de hipertensión arterial, dislipidemia, Enfermedad Pulmonar Obstructiva Crónica (EPOC) e IM hace 3 años acudió a urgencias refiriendo dolor torácico y disnea. Sus signos vitales fueron Tensión arterial (TA) 140/75 mmHg, Frecuencia cardiaca (FC) 88 lpm, Frecuencia respiratoria 22 cpm, Temperatura 37°C y Saturación de Oxígeno (SpO2) 91%. El examen físico mostró crepitantes bibasales. El electrocardiograma mostró una fibrosis anterior antigua sin otros cambios isquémicos (Figura I), y el laboratorio un NT-ProBNP de 1500 pg/dl, Troponinas ultrasensibles (TntUS) de 46 ng/L y un ácido láctico de 3,8 mmol/L. El ecocardiograma reveló acinesia septoapical e hipocinesia medioseptal con 50 % de fracción de eyección (Figura II). No se observaron otras particularidades en ningún otro método complementario inicial.

**Figura Nº 1.**
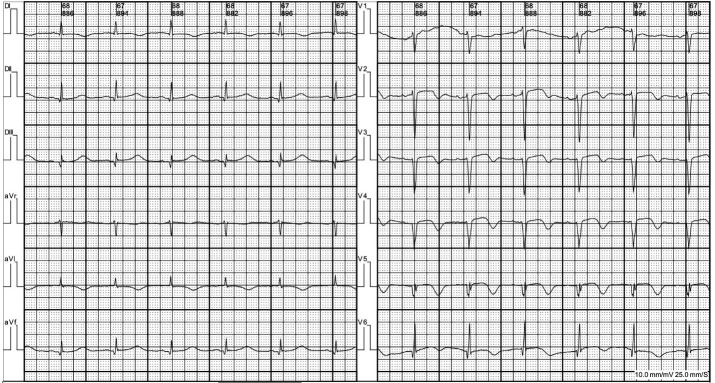
Electrocardiograma en el momento de la presentación en la UCC

**Figura Nº 2.**
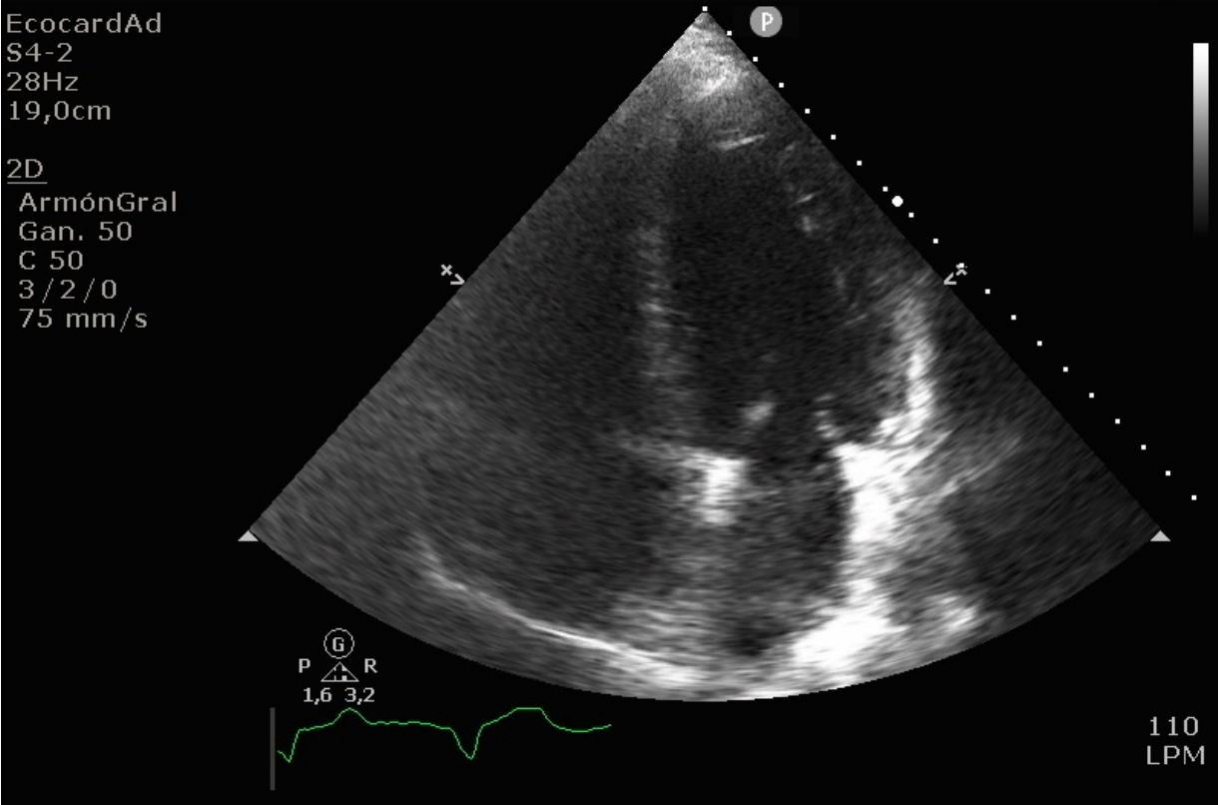
Ecocardiograma en el momento de la presentación en la Unidad Coronaria.

En la Unidad Coronaria (UCO) el paciente refirió exacerbación del dolor y se observó una taquiarritmia de QRS ancho con deterioro hemodinámico, por lo que se procedió a realizar Cardioversión Eléctrica con 200 Joules con éxito. La curva TntUS fue positiva (48/644/979) y el paciente evolucionó con Edema Pulmonar Agudo que requirió Ventilación No Invasiva e infusión de diuréticos y vasodilatadores.

El diagnóstico presuntivo fue Infarto de Miocardio sin Elevación del ST (IM-SEST) complicado con arritmia ventricular y Edema Agudo de pulmón (EAP).

La angiografía coronaria mostró la presencia de ectasias coronarias que afectaban tanto a la arteria Coronaria Derecha (Figura IIIa) como a ramas de las arterias Descendente Anterior y Circunfleja de la Coronaria Izquierda (Figura IIIb), sin lesiones obstructivas a tratar. pero con estenosis coronarias leves en tres vasos.

**Figura Nº 3.**
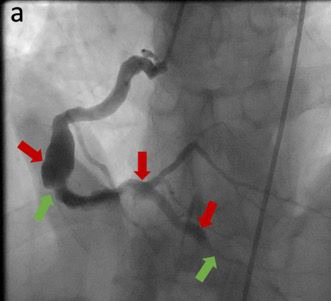

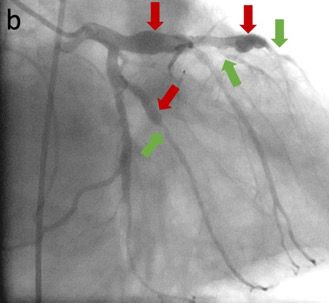
Imágenes de aterosclerosis y ectasia de las arterias coronarias derecha (a) e izquierda (b). Las flechas rojas muestran los cambios ectásicos y las flechas verdes los ateroscleróticos.

El paciente evolucionó favorablemente, con excelente respuesta al tratamiento médico y fue dado de alta a los tres días con Rivaroxabán 20 mg al día, Aspirina 100 mg al día, Rosuvastatina en dosis altas, Metoprolol y Losartán ajustados a valores de FC y TA.

## Discusión

Para el diagnóstico de MINOCA, la cuarta definición universal de IM requiere que se cumplan los criterios habituales y, además, no se demuestre una estenosis ≥50% en una arteria epicárdica principal ^1^ . MINOCA se puede subdividir en aterosclerosis nula o muy leve (0-30 % de estenosis) y aterosclerosis menor (30-49 % de estenosis) ^2^ .

Tanto la sociedad americana ^3^ como la europea ^4^ de cardiología han establecido estrategias diagnósticas en tres pasos en pacientes con sospecha de MINOCA. El primero es considerar el contexto clínico y excluir la embolia pulmonar y la sepsis, entre otros. El segundo paso es comprobar que no se trata de un verdadero Infarto asociado a Ectasia de arterias Coronarias (IM-EAC), reevaluando la coronariografía para detectar estenosis significativa u obstrucción de una rama lateral o menor. El tercer paso consiste en excluir causas no isquémicas de la presentación clínica y elevación de troponina, como miocarditis y síndrome de Takotsubo, mediante, por ejemplo, una Resonancia Magnética Cardiaca (RMNc) ^2^ . Desafortunadamente, nuestro paciente era claustrofóbico y se negó a someterse a una RMNc bajo anestesia general.

El electrocardiograma suele mostrar alteraciones del segmento ST, inversión de la onda T, o puede no presentar cambios, por lo tanto, los pacientes pueden presentar IM con Elevación del ST (IM-CEST) o un IM-SEST y cumplir con las definiciones de IM tipo 1 o tipo 2.

El IM tipo 2 es causado por un desequilibrio entre el suministro y la demanda de oxígeno del miocardio ^1^ . Nuestro paciente tenía varios mecanismos que podrían haber causado un IM tipo 2, ya sea por disminución del aporte de Oxígeno (O2) y aumento de la demanda, como ocurre con la arritmia ventricular rápida que padecía nuestro paciente. También presentaba edema pulmonar que, al disminuir el intercambio gaseoso, producía una disminución del aporte de O2 al miocardio.

A su vez, la EAC se define como una enfermedad difusa o dilatación local de una arteria coronaria epicárdica, con un diámetro que supera al menos 1,5 veces el segmento adyacente normal. Su prevalencia oscila entre el 0,3 y el 5% de todos los pacientes que acuden a realizarse una coronariografía ^5^ .

La EAC puede manifestarse clínicamente como Síndrome Coronario Agudo (SCA), angina de esfuerzo, isquemia silente, isquemia inducida por el ejercicio, disfunción microvascular, Muerte súbita Cardiaca (MSC), Miocardiopatía Isquémico Necrótica (MCD-IN), compresión de las arterias coronarias o estructuras cardíacas adyacentes y la trágica, aunque rara, ruptura con taponamiento cardíaco, y a menudo se reconoce ocasionalmente durante una angiografía coronaria. o una tomografía computarizada (TC). En pacientes con EAC, existen varios mecanismos posibles que conducen a SCA: 1, inestabilidad de la placa aterosclerótica con alta carga trombótica; 2, trombosis endoluminal debida a alteraciones del flujo sanguíneo y estasis, en ausencia de lesiones ateroscleróticas subyacentes; 3, embolización de trombo endoluminal distal; y 4, alteración de la perfusión miocárdica relacionada con flujo lento grave, que puede manifestarse clínicamente como SCA o angina de esfuerzo ^6^ .

Además, se ha encontrado que los pacientes con EAC son más jóvenes, tienen una enfermedad difusa que afecta a las tres ramas coronarias epicárdicas principales y tienen menos factores de riesgo cardiovascular tradicionales que aquellos con EAC mixta ^7^ , como nuestro paciente.

La ecografía intravascular se puede utilizar para confirmar la morfología y la dilatación de la luz. Markis et al propusieron una clasificación de EAC basada en la extensión de la afectación ectásica, siendo Tipo I: ectasia difusa de dos o tres vasos; Tipo II: enfermedad difusa en un solo vaso y localizada en otro vaso; Tipo III: enfermedad difusa en un vaso; y Tipo IV: ectasia localizada o segmentaria. Nuestro paciente tiene EAC tipoI ^8^ .

El tratamiento de la EAC es controvertido ^2^ . La progresión de la aterosclerosis y la extensión de la ectasia pueden estar relacionadas con el grado de estenosis. Por lo tanto, la prevención y el tratamiento de los cambios ateroscleróticos pueden tener más importancia clínica que abordar los cambios ectásicos. Las ectasias coronarias no variaron con el tiempo en nuestro paciente y sí la extensión aterosclerótica, aunque sin causar lesiones coronarias superiores al 50%.

La asociación entre MINOCA y MSC se describió previamente ^9^ . Ciliberti et al encontraron que la MSC generalmente ocurre en individuos jóvenes, a menudo en reposo o durante las actividades diarias y los síntomas cardíacos se informan en casi la mitad de los casos ^9^ . Nuestro paciente tuvo la suerte de estar en el hospital cuando desarrolló la arritmia que podría haber terminado en muerte en otro entorno. MINOCA tipo 3 se refiere a una MSC en pacientes con síntomas isquémicos, cambios en el ECG o fibrilación ventricular antes de que se puedan detectar biomarcadores en ausencia de estenosis coronarias, con identificación de IM en la autopsia ^10^ .

Finalmente, uno de nuestros objetivos fue relacionar fisiopatológicamente MINOCA con EAC. La patogenia de MINOCA se investigó recientemente ^11^ . Es importante destacar que la frecuencia de pacientes con EAC fue significativamente mayor en pacientes con MINOCA en comparación con aquellos con enfermedad obstructiva (22,8 % frente a 3,5 %, p < 0,001) y la frecuencia de MINOCA fue significativamente mayor en pacientes con EAC en comparación con pacientes sin EAC (35,9% vs. 6,4%, p < 0,001). La presencia de EAC fue un predictor independiente de MINOCA en pacientes con IM-SEST (OR 1.812, IC 95% 1.376 a 2.581, p < 0.001). La EAC puede considerarse un factor de riesgo y puede tener un papel en la fisiopatología de MINOCA, por lo tanto, la respuesta a nuestra pregunta sobre si EAC podría haber contribuido a MINOCA en nuestro paciente es SI. El mecanismo por el cual se presentó MINOCA en nuestro paciente pudo haber sido la formación de un trombo en las arterias ectásicas, el cual no se observó cuando se realizó la angiografía quizás porque estuvo anticoagulado 72 horas antes de la angiografía.

## Conclusión

Nuestro paciente presentó un MINOCA provocado por varios mecanismos fisiopatológicos en arterias coronarias ectásicas, que también podrían haber contribuido a ello. Son necesarios más estudios para el correcto diagnóstico y tratamiento, así como el abordaje de la EAC y su patogenia.
